# Readable and neutral? Reliability of crowdsourced misinformation debunking through linguistic and psycholinguistic cues

**DOI:** 10.3389/fpsyg.2024.1478176

**Published:** 2024-11-13

**Authors:** Mengni Yao, Sha Tian, Wenming Zhong

**Affiliations:** ^1^College of Foreign Languages, Nankai University, Tianjin, China; ^2^School of Foreign Languages, Central South University, Changsha, Hunan, China

**Keywords:** misinformation debunking, crowdsourcing, Community Notes, LIWC, linguistic features

## Abstract

**Background:**

In the face of the proliferation of misinformation during the COVID-19 pandemic, crowdsourced debunking has surfaced as a counter-infodemic measure to complement efforts from professionals and regular individuals. In 2021, X (formerly Twitter) initiated its community-driven fact-checking program, named Community Notes (formerly Birdwatch). This program allows users to create contextual and corrective notes for misleading posts and rate the helpfulness of others' contributions. The effectiveness of the platform has been preliminarily verified, but mixed findings on reliability indicate the need for further research.

**Objective:**

The study aims to assess the reliability of Community Notes by comparing the readability and language neutrality of helpful and unhelpful notes.

**Methods:**

A total of 7,705 helpful notes and 2,091 unhelpful notes spanning from January 20, 2021, to May 30, 2023 were collected. Measures of reading ease, analytical thinking, affect and authenticity were derived by means of Wordless and Linguistic Inquiry and Word Count (LIWC). Subsequently, the non-parametric Mann–Whitney *U*-test was employed to evaluate the differences between the helpful and unhelpful groups.

**Results:**

Both groups of notes are easy to read with no notable difference. Helpful notes show significantly greater logical thinking, authenticity, and emotional restraint than unhelpful ones. As such, the reliability of Community Notes is validated in terms of readability and neutrality. Nevertheless, the prevalence of prepared, negative and swear language in unhelpful notes indicates the manipulative and abusive attempts on the platform. The wide value range in the unhelpful group and overall limited consensus on note helpfulness also suggest the complex information ecology within the crowdsourced platform, highlighting the necessity of further guidance and management.

**Conclusion:**

Based on the statistical analysis of the linguistic and psycholinguistic characteristics, the study validated the reliability of Community Notes and identified room for improvement. Future endeavors could explore the psychological motivations underlying volunteering, gaming, or even manipulative behaviors, enhance the crowdsourced debunking system and integrate it with broader efforts in infodemic management.

## 1 Introduction

Misinformation pervades a multitude of topical domains, spanning from health discourses to political narratives, and rapidly disseminates through diverse media channels (Southwell et al., 2018). Individuals, due to psychological and sociological predispositions, are susceptible to misleading information (Ecker et al., 2022) and easily affected by the inflammatory and sensational language (Rashkin et al., 2017). During the COVID-19 pandemic, the rampant dissemination of heterogeneous and unverified information impeded interpersonal and intercultural communication, further exacerbating societal divisions (Chong et al., 2022; Liu and Cheung, 2023). With the recent advance of generative artificial intelligence (AI), large language models enable the rapid and extensive generation of human-like and personalized misinformation, exacerbating the complexity of the issue (Kreps et al., 2022). Given this, misinformation debunking, the pillar of infodemic management (Eysenbach, 2020), has emerged as a critical focus within the academic circle.

Three prevalent fact-checking practices can be identified from the perspective of implementation timing. Firstly, proactive measures such as early warnings and educational interventions (Guess et al., 2020), rooted in the psychological theory of inoculation, can preemptively cultivate and fortify users' resilience to misinformation (Jiang et al., 2022; Lewandowsky and Van Der Linden, 2021). Nevertheless, prebunking, as a tricky and long-term task, has shown to be less efficacious than reactive debunking (Tay et al., 2022). The second approach involves training a classification model by distinct characteristics of fake information and subsequently applying it to real scenarios, so as to monitor, label, down-rank or even remove false claims and suppress their proliferation if the situation permits. The misinformation classification methods are hindered by the scarcity of fine-grained, pre-annotated and up-to-date training data (Carrasco-Farré, 2022) and experience a performance drop when identifying human-generated misinformation compared to AI-generated misinformation (Zhou et al., 2023), indicating room for improvement (Aïmeur et al., 2023). The last line of work addresses misinformation after its emergence, utilizing professional, layperson-based and crowdsourced efforts. News personnel and domain experts can provide informative and authoritative content. However, there are inherent limitations of professional fact-checking, particularly regarding coverage and speed (Martel et al., 2024). In contrast, the feasibility of layperson-based debunking has been preliminarily validated (Bhuiyan et al., 2020; Pennycook and Rand, 2019; Wineburg and McGrew, 2019), implying the promise of organized public engagement as a supplementary strategy.

Community Notes represents X's innovative fact-checking initiative, designed to swiftly and properly combat misinformation through extensive public participation. At the beginning of 2021, X introduced Community Notes, previously branded as Birdwatch. The platform was initially accessible solely to pilot users within the U.S., and gradually expanded its reach to moderators from other regions after December 2022. Within this community-driven framework, a set of rules has been designed to build a well-structured and healthy information ecosystem, ensuring that informative notes contributed by users can be attached to suspicious tweets. Despite Community Notes' efforts in platform governance and the impressive claims for its transparency, challenges and risks such as data poisoning, algorithmic exploitation, and coordinated malicious attacks persist (Benjamin, 2021). It is necessary to assess the reliability of Community Notes and explore ways to enhance crowdsourced debunking.

The paper aims to evaluate the reliability of misinformation debunking on Community Notes in terms of readability and neutrality. “Easy to understand” and “neutral language” are outlined as helpful attributes according to the official guideline,^1^ and also recognized as effective language patterns in similar contexts. Considering that language use has been demonstrated to be informative by linguistics research, particularly in psychology (Pennebaker and King, 1999), these two variables are adopted to examine platform priorities in regulating online content and scrutinize whether helpful voices are amplified or marginalized. The study poses two research questions (RQs):

RQ1: How is the reliability of Community Notes in terms of readability?

RQ2: How is the reliability of Community Notes in terms of neutrality?

To address the research questions, the study collected helpful and unhelpful notes from the open Community Notes dataset, and analyzed linguistic and psycholinguistic characteristics of two groups using Wordless and Linguistic Inquiry and Word Count (LIWC). The helpful and unhelpful groups display equal levels of readability; moreover, the former demonstrates significantly greater logical thinking, authenticity, and emotional restraint. These findings validate the reliability of Community Notes. However, the unhelpful group shows a notable presence of prepared, negative, and swear language, along with a wide range of values in the measures. Additionally, the overall consensus on note helpfulness is limited. These indicate areas for improvement in the crowdsourcing management system. The study contributes to the understanding of reliability and potential challenges of crowdsourced debunking and provides insight to platform management and its integration into broader efforts.

## 2 Literature review

### 2.1 Crowdsourced misinformation debunking

Professional and non-professional debunkers have employed various methods to dismantle and mitigate the impact of fake information, achieving certain degrees of success. Debunking refers to the provision of corrective information to establish that the previous message is incorrect or misleading. This is a complex process where different cognitive frames compete and collide with each other. Debunking practices from professionals, such as those in the governmental sector, public health, journalism and specialized fact-checking organizations, have long been an integral part of infodemic management on social media. Authorities and experts are considered effective in enhancing the public's awareness of crisis severity (Van der Meer and Jin, 2020) and maintaining the overall stability of the public opinion (Zhong and Yao, 2023). However, studies have indicated that official sources are also criticized for being slow, obsolescent, invisible, thereby leading to limited and delayed dissemination and even fostering mistrust (Micallef et al., 2020). In view of this, recent studies on online misinformation have highlighted the potential for regular people to leverage their advantages in countering misleading information. The capability of non-experts to discern between highly credible and less credible news sources (Bhuiyan et al., 2020; Pennycook and Rand, 2019), as well as the verification procedures employed by individuals with different educational backgrounds and identities (Wineburg and McGrew, 2019) has been validated as prerequisite. Additionally, individuals are willing to share the information which they have personally searched for and verified (Li and Xiao, 2022).

Crowdsourced debunking, a new form of non-professional debunking, has emerged and is expected to play a complementary role with faster speed, greater volume and more systematic management. Crowdsourcing allows individuals or organizations to outsource tasks to specific population of actors, akin to the operational mode of Wikipedia and Stack Overflow. Its advantages stem from low cost, high efficiency, anonymity, and a strong user-platform connection. Particularly, the accumulation of user knowledge could potentially be closer to the truth than individual efforts and even those of experts (Bhuiyan et al., 2020; Woolley et al., 2010). There are two types of crowdsourced fact-checking: One involves recruiting ordinary individuals from crowdsourcing marketplaces such as Amazon Mechanical Turk to evaluate and annotate the accuracy of content, commonly used as an experimental method in academic research (Saeed et al., 2022); the second one motivates the public to collaboratively and voluntarily generate novel knowledge and insights in the form of fact-checks, which is the focus of this paper.

There are some attempts in this regard. In the experimental context, Pinto et al. (2019) proposed the fact-checking workflow, which can be sustained and overseen by the crowd itself, and advocated for the utilization of a diverse workforce and resources to increase the volume and reach of refutation efforts. In practical settings, Cofacts, a community-driven fact-checking platform in Taiwan, China, has captivated researchers. Zhao and Naaman (2023a) found that it performed on par with two professional fact-checking sites in terms of veracity and viability, while surpassing them in velocity. Zhao and Naaman (2023b) further observed that Cofacts' sustainability was intrinsically linked to Taiwan's dynamic civic tech culture and longstanding tradition of crowdsourcing activism. The findings indicate that while crowdsourced debunking holds substantial promise, it demands considerable labor and continuous engagement.

### 2.2 Operating mechanism of Community Notes

During the COVID-19 pandemic, X leveraged its advantages of large user base and well-established interactive frameworks to launch the crowdsourcing platform, marking a fresh attempt to combat misinformation.

On Community Notes, users are encouraged to assess the veracity of suspicious tweets and provide contextual evidence, termed notes. Individuals who engage in the process are referred to as contributors or debunkers. They constitute a voluntary community in which a stringent mechanism regulates user participation. As far as a user is concerned, newcomers start with an initial Rating Impact score of zero and must consistently rate submitted notes on the level of helpfulness to gain the eligibility for writing notes themselves. Subsequently, contributors can accumulate their Writing and Rating Impact scores by producing helpful notes and evaluating ones written by others. However, their writing privileges may be temporarily suspended by the system once their notes are frequently deemed unhelpful. That is to say, the dynamic system generates the reputation impact based on users' track records, and in turn influences the qualification in subsequent periods (Pröllochs, 2022).

Regarding notes, if a consensus can be reached among a broad and diverse group of contributors, the note will be transferred to X and displayed directly below the suspicious tweet for all X users. The note status would be updated as new ratings are received until it is locked after 2 weeks. This bridging-based ranking system, designed to make it more difficult for accounts to spam the system with low-quality ratings, allows for the better identification of higher-quality content (Wojcik et al., 2022).

Additionally, Community Notes has implemented several measures to enhance the system. For instance, it encourages individuals with diverse perspectives to participate in rating. When establishing what constitutes different perspectives, Community Notes does not consider demographics such as location, gender, or political affiliation, nor does it use data from X as indicators. Instead, it objectively focuses on how individuals rated notes in the past. All of these operational mechanisms are supported by rigorous and complex algorithms and Community Notes is continuously updating rules.

The emergence of Community Notes has raised concerns about its effectiveness and reliability. Effectiveness, a common issue in the misinformation field, focuses on the final outcome of rebuttal. It aims to examine the influence of corrective messages on receivers, such as spread curve of misinformation and changes in receivers' conception or behaviors (Pröllochs, 2022). Since people often fall for misinformation due to a lack of careful reasoning, relevant knowledge, and reliance on heuristics such as familiarity, corrective notes are expected to help them discern truth (Pennycook and Rand, 2021). As for reliability, in the context of expert and layperson-based debunking, it is often associated with terms such as accuracy, quality, credibility and trustworthiness (Adams, 2006). However, social media platforms as black boxes are often suspected of manipulation and abusive use (Ferrara et al., 2020). Hence, in this study, the reliability of crowdsourced misinformation debunking is defined as platform's ability to foster a transparent and healthy information environment while providing information beneficial for debunking as much as possible. Crowdsourcing requires proper management; otherwise, each step in the process may jeopardize reliability. Benjamin (2021) outlined a set of potential risks on Community Notes. To name a few, are there instances of fake or sock puppet accounts? Is there any coordinated manipulation attempted to oversee, filter, and regulate user access to notes? Is there indication that contributors' political party affiliations might impact their personal opinions and value judgments, consequently contributing to polarization?

In general, Community Notes represents a new effort in crowdsourced debunking, and there is limited research on it.

### 2.3 Evaluation of Community Notes

For this emerging platform, some studies have conducted preliminary research on its effectiveness and reliability. Regarding its effectiveness, research indicated that misleading tweets accompanied by notes tended to spread less virally compared with ones without such annotations (Drolsbach and Pröllochs, 2023). Furthermore, individuals exposed to corrective notes exhibited a 25–34% lower likelihood of liking, replying to or resharing misinformation compared to those who were not, suggesting observable changes in user behavior (Wojcik et al., 2022). Compared with professional fact-checking, Community Notes demonstrated relatively good performance as well. Pilarski et al. (2024) analyzed the differences between Community Notes and Snoping, a conversational fact-checking approach primarily built upon professional judgments. Their study revealed that note contributors and Snopers paid attention to different tweets, thereby facilitating the fact-checking coverage across a broad spectrum of social media posts. Meanwhile, those overlapping also demonstrated a notable level of consensus in the veracity. Nevertheless, Chuai et al. (2023) also pointed out that Community Notes may not act swiftly enough to curb the dissemination of misinformation during its initial and highly contagious phase. Overall, the platform's effectiveness appears promising at present, albeit with some response delay.

As for reliability, few concerns have been addressed in this regard. The quality and relevance of evidence presented in notes have received significant academic attention. Evidence like URLs and citations is a crucial component frequently integrated into corrective messages (He et al., 2023), proving valuable in rectifying misperceptions across social media platforms (Vraga and Bode, 2018). Saeed et al. (2022) delved into the sources of evidence mentioned in the notes and assessed their reliability. The study collected 12,909 links from the Community Notes dataset and extracted a total of 2,014 domains. Through manual review by journalists, it was found that note links upvoted as high quality by Community Notes users, consistently garnered high journalist scores. Allen et al. (2024) also focused on the quality of citations. They double rated the credibility of sources based on three tiers, high, moderate and low. It is found that only 7% notes cited low credibility sources, such as blogs or tabloids. In addition to manual review of evidence credibility by professionals, Simpson (2022) adopted Kullback–Leibler divergence and the document probability distributions to investigate the relevance of notes and tweets. There was a significant topic overlap between tweets and notes with higher note ratings. Therefore, the reliability of Community Notes has been preliminarily verified through evidence use.

Additionally, some scholars evaluated the reliability of Community Notes based on its built-in voting and ranking system. Ovadya (2022) held that the platform surpassed many engagement-based ranking systems. However, Allen et al. (2022) investigated the influence of partisanship among participants and discovered that they exhibited a tendency to assign negative annotations to tweets from those with opposing political affiliations and perceive their annotations as less helpful. Mujumdar and Kumar (2021) also identified the loophole, that is a small number of fake accounts could elevate any random note to a top-ranked status. To address this, they introduced a novel reputation system called HawkEye. The system incorporates a cold-start-aware graph-based recursive algorithm and evaluates the intrinsic quality of user trust, note credibility, and tweet accuracy, in order to mitigate the vulnerability of Community Notes to adversarial attacks.

The effectiveness of Community Notes has received certain agreement. However, the ongoing controversy regarding its reliability underscores the urgent need for further research.

### 2.4 Readability and neutrality as helpful attributes

Note writing and voting requirements officially outlined by Community Notes provide insight into establishing measures of reliability. Community Notes has delineated note requirements in its user guidelines and instructed all contributors to write and rate notes as helpful or unhelpful accordingly. They list the following helpful attributes:

(1) Cites high-quality sources;
(2) Easy to understand;
(3) Directly addresses the post's claim;
(4) Provides important context;
(5) Neutral or unbiased language.

The above requirements guide the entire process of note creation and ranking. Hence, these can be adopted as reliability measures to explore whether users write and vote helpful notes as required and whether Community Notes amplify the helpful voice on X. The indicators include two aspects. One pertains to what notes convey, which corresponds to the first, third and fourth attributes, dealing with the credibility, relevance and coverage of notes, respectively; another concerns how notes are conveyed, reflected in the second and fourth attributes. These refer to the readability and neutrality of notes. Given that the former aspect has been extensively studied, as summarized above, this paper specifically examines the reliability of Community Notes in terms of readability and neutrality.

#### 2.4.1 Readability

Readability refers to “the ease of understanding or comprehension due to the style of writing” (Klare, 1963), which can be derived by readability formulas with various purposes and settings (DuBay, 2004). Reading ease is the determinant of whether receivers process a debunking message with the central route. Receivers must possess the necessary cognitive capacity and linguistic comprehension. Once the language or message complexity exceeds their cognitive capabilities, individuals are less inclined to engage in extensive elaboration (Petty et al., 1986) and are likely to generate negative judgments toward corrective messages (Schwarz, 1998). Wang et al. (2022) examined the impact of the readability on the acceptance of rebuttal texts on Sina Weibo, often called “Chinese Twitter”. Using the frequency of common characters in the Chinese dictionary to evaluate readability, the study indicated that greater readability had a positive influence on the public's acceptance of the rebuttal. Furthermore, corrective messages often involve specialized terms and knowledge. The manner in which new scientific and technological advancements, and evolving epidemiological information, are presented is significant (Daraz et al., 2018). A digestible format not only builds trust among recipients but also facilitates the dissemination on social media, especially supporting highly vulnerable refugee, immigrant, and migrant communities with limited language proficiency (Feinberg et al., 2023).

This underscores the importance of readability in effective persuasion and refutation. Therefore, to address the first research question (RQ1), the study hypothesizes the following,

**H1:** Helpful notes are more readable than the unhelpful ones.

#### 2.4.2 Neutrality

Another crucial attribute is neutral language, which focuses on the way users presenting note here. Content neutrality, like no selection, omission or exaggeration of facts (Hamborg et al., 2019), are conscious, controllable, and easy to report (Wilson et al., 2000). By contrast, language bias are implicit and unconscious. It is frequently associated with specific linguistic features, such as the abstraction level of words based on the linguistic category model (Maass et al., 1989), hedges, subjective intensifiers (Recasens et al., 2013), referring expressions (Cheung, 2014), direct and reported speech (Cheung, 2012), lack of logical and analytical thinking (Huang and Wang, 2022; Vraga et al., 2019), as well as praising, selling, inflammatory, or hateful expressions (Recasens et al., 2013) and so on. Bias detection can be achieved by natural language processing like LIWC (Hube and Fetahu, 2018; Niven and Kao, 2020), and machine learning techniques (Spinde et al., 2023; Vallejo et al., 2024).

The importance of neutral language has been emphasized in complex information dissemination settings. It is found that neutrally phrased language is crucial to avoid stirring disagreement among parties in Wikipedia, news media and political debates (Hamborg et al., 2019; Hube and Fetahu, 2019; Iyyer et al., 2014). Similarly, the issue has also been examined in the field of misinformation debunking from different angles. Since there are a lot of causal explanations in debunking, logic-based corrections can effectively reduce the credibility of misinformation (Vraga et al., 2019) and wield greater influence in changing attitudes and behavioral intentions when compared with the narrative-based approach (Huang and Wang, 2022). Furthermore, studies also validated the association between emotion and bias. Although Cappella et al. (2015) looked for the possibilities of using emotional messages to counteract the emotional aspect of belief echoes, emotionally charged statements especially swear words have been proven unsuitable for social media platforms (Vo and Lee, 2019), due to their tendency to provoke stronger emotional contagion and conflicts (Clore and Huntsinger, 2007).

There is still a vacancy in the language neutrality of corrective messages on Community Notes. Only Pröllochs (2022) found notes were more negative toward misleading tweets than not misleading ones, necessitating further studies. Given the expectation for neutral notes on the platform and the observed gap, the study proposes the following hypothesis,

**H2:** Helpful notes are more neutral than the unhelpful ones.

## 3 Methodology

The study gathered the open-sourced notes voted as helpful and unhelpful by users and evaluated the reliability of Community Notes through quantitative features grounded in linguistic and psychological sciences.

### 3.1 Data collection and preprocessing

#### 3.1.1 Data collection

First, four separate files were downloaded, i.e., Notes, Ratings, Note Status History, and User Enrollment, from the Community Notes' public data page^2^ on June 25, 2023. Second, these tables were merged into a unified dataset that encompasses note ID, creation time, note content, and locked status. Since the focus is on notes that reached a consensus among a sufficient number of raters and were assigned locked statuses after a period of 2 weeks, other information like rating history, rating reasons were not taken into consideration. Third, hundreds of thousands of notes labeled as NEEDS_MORE_RATINGS and a few written in languages other than English were removed. Consequently, a total of 7,705 helpful notes and 2,091 not helpful notes were collected, spanning from January 20, 2021 to May 30, 2023.

It is noteworthy that if writers delete notes and ratings, the metadata would be documented in the file of Note Status History, but the textual content of the notes is no longer officially available. Moreover, Community Notes invites public and scholarly scrutiny of its performance by making all of the data accessible and downloadable online. Therefore, the study using public data was exempted from ethical review.

#### 3.1.2 Data preprocessing

External links and converted escape characters were excluded, such as &quot; and &amp into normal ones, because these would influence the results of linguistic features and citation sources are not the focus in this paper.

### 3.2 Measures

The assessment of Flesch Reading Ease for readability was conducted using Wordless. LIWC was also employed to identify three relevant characteristics of neutrality: analytical thinking, authenticity, and affect.

#### 3.2.1 Readability

##### 3.2.1.1 Wordless

Wordless is an integrated corpus tool that allows users to explore prevalent linguistic features within textual data, such as readability, counts, lengths, keywords, concordance and collocation (Ye, 2024). The 3.4.0 version was adopted.

##### 3.2.1.2 Flesch Reading Ease

Wordless was utilized to obtain the Flesch Reading Ease score and assess the readability of the note. Compared with readability measures that are tailored for specific domains, impose basic thresholds for word count, or rely on fixed dictionaries, the Flesch Reading Ease is flexible and comprehensive. Therefore, it is highly recommended across all sectors and disciplines (DuBay, 2004). Flesch scores primarily consider two factors: the average number of syllables per word and the average number of words per sentence (Flesch, 1949). For the Flesch Reading Ease, a higher value indicates easier readability, contrary to the majority of readability formulas where lower value signifies easier readability. Generally, readability values fall within the 0–100 range under normal circumstances. However, due to the computational mechanism of the formula, the values may exceed this range if a text is either too simple or too complex.

#### 3.2.2 Neutrality

##### 3.2.2.1 LIWC

Linguistic Inquiry and Word Count is a lexicon and rule-based software designed to analyze psychological and emotional constructs in texts. Language patterns have the strong diagnostic power for style and people's underlying social and psychological world (Tausczik and Pennebaker, 2010). Based on this, LIWC builds up an internally consistent language dictionary with enhanced psychometric properties. It functions by searching each word in a text with the dictionary, and quantifying the percentage of matched words assigned for different features (Boyd et al., 2022).

In terms of applicability, the software has demonstrated effectiveness in quantifying, understanding, and elucidating the biased statement in news media (Niven and Kao, 2020), crowdsourced knowledge generation (Hube and Fetahu, 2018) and professional misinformation debunking (Vo and Lee, 2019). Furthermore, the current iteration of LIWC is no longer constrained by text length. With the inclusion of emoticons, short phrases, and netspeak language, LIWC can generate reliable and accurate results when analyzing tweets, Facebook posts, and SMS-like modes of communication (Boyd et al., 2022).

LIWC-22 was employed to calculate scores for analytical thinking, authenticity, and affect in both helpful and unhelpful notes.

##### 3.2.2.2 Analytical thinking

Logic-based corrections are found effective in reducing the credibility of misinformation and changing the attitudes and behavioral intentions of recipients (Vraga et al., 2019; Huang and Wang, 2022). Hence, *Analytic*, the summary feature in LIWC-22 was adopted to capture the extent to which individuals employ words indicative of formal, logical, and hierarchical thinking patterns. The analytical thinking formula encompasses various categories of words, including articles, prepositions, pronouns, auxiliary verbs, conjunctions, adverbs, and negations (Ta et al., 2022). For instance, connectives are vital for conveying implicit interclausal relations and the underlying logic (Cheung, 2009; Li et al., 2022).

##### 3.2.2.3 Authenticity

*Authentic*, also a summary feature in LIWC-22, refers to the extent to which individuals communicate in alignment with their true selves (Newman and Dhar, 2014). That is to say, authenticity are irrelevant with the exact content or whether it is true or false, but rather with perceived genuineness. Specifically, the authenticity formula incorporates some elements common in sincere speech, such as first-person pronouns and relativity words and present tense (Fox and Royne Stafford, 2021). This definition was applied in the study. Authenticity can examine the extent to which users on Community Notes freely and naturally express their beliefs and values. This is particularly crucial for identifying whether notes have been prepared, filtered, or manipulated due to political and social inhibitions (Allen et al., 2022; Benjamin, 2021).

##### 3.2.2.4 Affect

In light of the fact that emotionally charged statements readily provoke stronger emotional contagion on social media (Clore and Huntsinger, 2007; Vo and Lee, 2019), *Affect* was adopted to investigate whether helpful notes exhibit greater affective restraint. Unlike the aforementioned summary features, *Affect* comprises several subordinate categories: *tone* (emotional tone), *emotion_pos* (positive emotion), *emo_neg* (negative emotion), *emo_anx* (anxiety), *emo_anger* (anger), *emo_sad* (sadness), and *swear* (swear words), among others. Good, love, happy, hope and other emotion-related words, word stems, phrases, and emoticons are included in the LIWC dictionary for calculation (Boyd et al., 2022).

SPSSAU was used to conduct the statistical analysis. Given the non-normal distribution of the data, the non-parametric Mann–Whitney *U*-test was employed to examine the statistical differences in the above measures between the helpful and unhelpful groups.

## 4 Results

### 4.1 Reading ease for both groups

**Figure 1:**
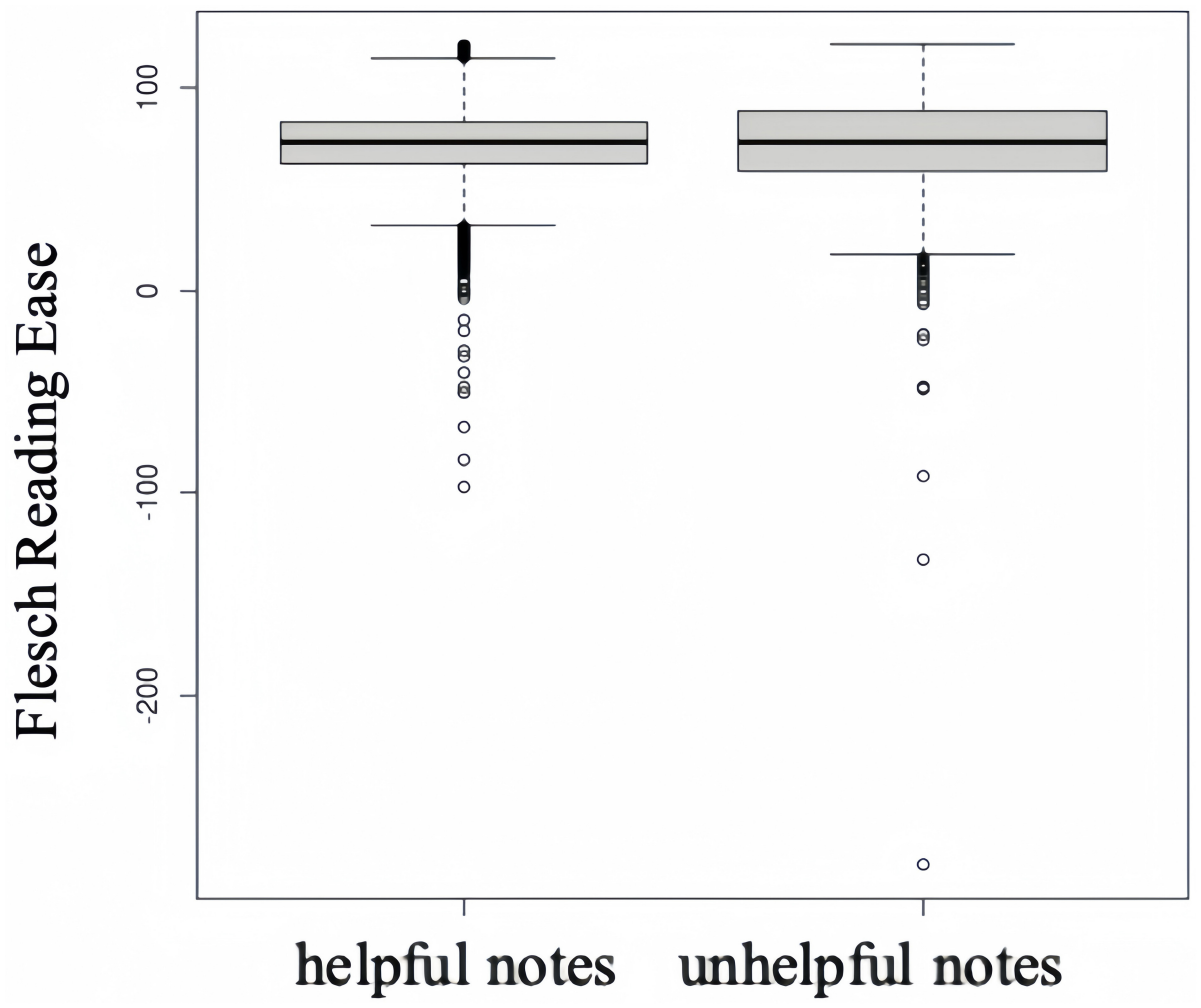
Readability of helpful and unhelpful notes.

In what follows, results of the non-parametric Mann–Whitney *U*-test are presented with median, 1st quartile, 3rd quartile, *z*-value and *p*-value. The study initially investigates the difference in readability between helpful and unhelpful notes for RQ1. Figure 1 illustrates the distribution of Flesch reading ease values for two distinct groups. The median readability value is 73.483 (IQR = 62.6–83.4) for helpful notes and 73.172 (IQR = 59.2–87.9) for the unhelpful, indicating that helpful notes are slightly easier to understand than unhelpful ones. A reading score of 70–80 corresponds to a 7th-grade reading level, which means notes from both groups were easy enough for 12–13-year-olds to process. However, the Mann-Whitney *U*-test yields a *z*-value of −0.827, suggesting no significant difference in readability between two groups (*p* = 0.408). Thus, H1 is not supported.

### 4.2 Unbiased language in the helpful group

**Figure 2:**
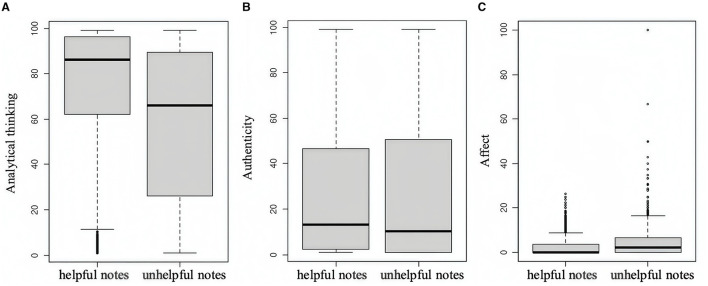
**(A–C)** Neutrality of helpful and unhelpful notes.

For RQ2, the results of three neutrality measures are shown in Figure 2. Regarding analytical thinking, the median value for helpful notes stands at 86.153 (IQR = 62.1–96.4), while for unhelpful notes it is 66.040 (IQR = 26.1–89.5), as depicted in Figure 2A. The difference observed is statistically significant (*z* = −20.685, *p* < 0.000), emphasizing that helpful notes involve more analytical thinking than not helpful notes. Furthermore, two groups also vary in authenticity, which is supposed to reflect perceived honesty and genuineness. Helpful notes (Med =13.332, IQR = 2.4–46.6) are far more authentic than not helpful ones (Med =10.181, IQR = 1.0–50.4) with a statistically significant difference from each other (*z* = −3.976, *p* < 0.000). In terms of affect, notable differences are observed, as shown by the boxplot in Figure 2C. Helpful notes (Med = 0.000, IQR = 0.0–3.6) show less affect, while another group (Med =2.174, IQR = 0.0–6.7) exhibits stronger sentiment and emotion (*z* = −13.07, *p* < 0.000).

**Figure 3:**
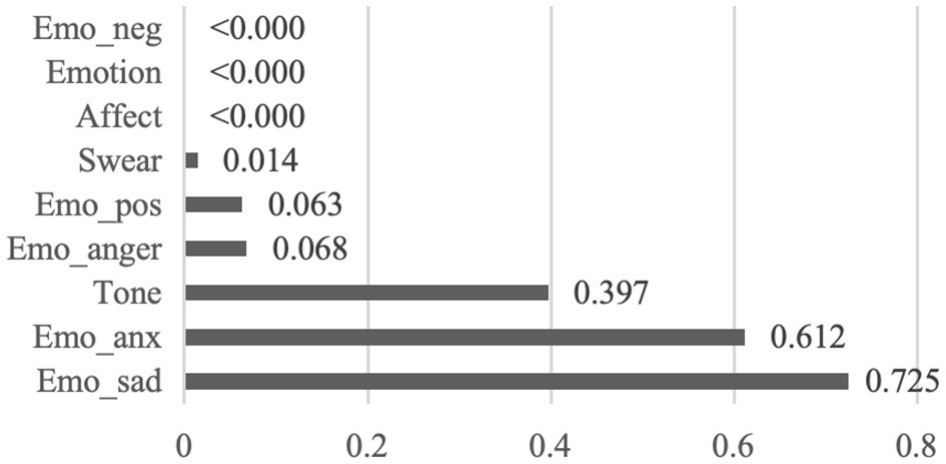
P-values for the sub-categories of affect between helpful and unhelpful notes.

Figure 3 illustrates the closer examination of sub-categories of affect. Two groups show no statistically significant difference in tone (*p* = 0.397), emo_pos (*p* = 0.063), emo_anx (*p* = 0.612), emo_anger (*p* = 0.068) and emo_sad (*p* = 0.725). In contrast, they differ in emo_neg (*p* < 0.000) and swear (*p* = 0.014), indicating that unhelpful notes contain more negative emotion and swear words. Overall, analytical thinking, authenticity and affect are crucial predictors of system reliability within the context of misinformation debunking. H2 is supported.

## 5 Discussion

The study investigates the reliability of crowdsourced debunking in terms of readability and neutrality. The results indicate that both helpful and unhelpful group exhibit ease of comprehension, yet the former distinguish itself through its stronger logical thinking, enhanced authenticity and diminished emotion relative to the latter. The user-endorsed helpful notes align with the note writing and voting guidelines established by Community Notes, underscoring the reliability of crowdsourced debunking in these two aspects. The analysis and insights behind the results are further elaborated below.

Firstly, the reliability of the platform is validated as the helpful notes are easily understood and more neutral than the unhelpful group. With respect to readability, the statistic analysis reveals that there is no discernible difference in the Flesch Reading Ease scores between the helpful and unhelpful notes. Regardless of this, both are readable enough for 12–13-year-olds to understand, in accordance with the official requirements, thereby still verifying the reliability. This suggests that, for one thing, both groups adhere to the linguistic norms of Community Notes. For another, this may also be attributed to the plain language conventions on the Internet. In any case, this ensures that information is accessible to a wide population with different levels of language proficiency (Feinberg et al., 2023).

In terms of neutrality, there is the disparity between the two groups. To begin with, helpful notes demonstrate a higher degree of analytical language compared with unhelpful notes, displaying greater logical coherence and a less narrative style. Notably, the research team of LIWC analyzed test corpora of blogs, X and *New York Times*, presenting the mean value of analytical thinking as 38.70, 42.86, and 87.62 respectively (Boyd et al., 2022). For helpful notes, the median value of analytical thinking stands at 86.153 (IQR = 62.1–96.4). Although the statistical comparison is not feasible due to the difference in the mean and median, this suggests that the level of analytical thinking in helpful notes approaches that of news writing and surpasses most social media discourse. This highlights that non-professionals engage in a slow and deliberate information processing route, thereby maximizing their efficacy in executing the debunking task (Stanovich, 2009). In addition, helpful notes are more embedded with users' mental processes in an unconscious and spontaneous manner. The self-representation aligns with the established criteria. Lastly, the relatively low affect value of the helpful notes also indicates a restrained tone and emotional expression. On the whole, the helpful group shows good performance in terms of readability and neutrality, thereby justifying the platform's reliability.

Secondly, some measures pertaining to unhelpful notes indicate a discernible tendency among users to post malicious content. This is consistent with previous studies that show significant concerns over the dishonest and malicious attempts on the platform (Benjamin, 2021). According to the definition proposed by LIWC, lower values in authenticity for unhelpful notes mean a greater degree of preparedness or social caution, thereby implying the presence of guarded positions and malicious intents behind them. In addition, although both groups exhibit restraint in tone and most types of emotions with no significant differences, there is an exception. The unhelpful notes employ a higher frequency of negatively emotional language and swear expressions, which is in line with the observed negative correlation between emotion and analytical thinking (Clore and Huntsinger, 2007). One of the plausible explanations for the prevalence of negative emotions could be unconscious yet harmful behavior. Emotions encompass a subjective array of feelings, cognitive assessments of situations and physiological arousal (Nabi and Oliver, 2009). Jiang and Wilson (2018) identified that misinformation, particularly when infused with inflammatory content and a sensational writing style, would affect the emotional markers in comments, such as using extensive emoji and swear words. As a result, critically engaging with an abusive tweet might lead to a note being perceived as hateful. This aligns with the extant finding that notes are more negative toward misleading tweets than accurate ones (Pröllochs, 2022). Alternatively, this phenomenon may also be attributed to the deliberate leverage of negative emotional language to elicit strong public reactions or even systematically target at specific groups. Such behavior parallels the motivational factors behind malicious rating, as both stem from conflicting values or beliefs (Allen et al., 2022). In this way, the abuse and weaponization of language are indeed significant issues on Community Notes.

Thirdly, the value ranges for measures within the unhelpful group are too large and users seldom reach consensus on helpfulness of notes, indicating the need to enhance the efficiency and management standards of the platform. The number of unhelpful notes is fewer than that of helpful ones, but the ranges of values are broader across all measures. Numerous outliers are also evident in the boxplot analyses. On the one hand, the reliability of the writing and ranking system is validated, as evidenced by the tendency for helpful notes to demonstrate superior performance when contrasted with unhelpful ones. On the other hand, it reveals that community-driven content is a mixed bag with varying shades. Users may lack a sufficiently clear understanding of the debunking mission or even harbor undisclosed intentions. Furthermore, from a broader perspective, the platform has been flooded with hundreds of thousands of notes since its pilot launch in the U.S. and subsequent global rollout. This highlights the advantages inherent in a crowdsourced approach over the professional one in terms of volume and velocity (Zhao and Naaman, 2023a). However, fewer than 10,000 reached a consensus on helpfulness, with 7,705 classified helpful. That is to say, most attempts from users failed. This supports the notion that Community Notes is too slow to react in the early stage of misinformation dissemination (Chuai et al., 2023). Given that tweets typically reach half of their total impressions within ~80 min (Pfeffer et al., 2023), if notes couldn't be helpful enough to be visible on X in a short time, the effectiveness might be hindered for the time delay. These two phenomena partially support the skepticism regarding the effectiveness of crowdsourcing for dispelling rumors and raise concerns about its managerial competence.

The research contributes to the scant crowdsourced debunking literature by closely examining and comparing four linguistic and psychological measures of upvoted notes on Community Notes. Considering that the coexistence of earnest contributions and malicious attempts on the platform is observed, future studies could delve into the psychological factors shaping crowdsourced debunking, including exploring users' motivations to volunteering or gaming the system, and discussing the potential for coordinated campaigns to ideologically or psychologically manipulate Community Notes. At a practical level, the platform can taxonomize and prioritize risks associated with crowdsourced debunking by evaluating factors such as likelihood and severity, and subsequently establish a more specific and rigorous messaging guideline and assessment model. For example, showing respect to others. If users could focus on the false tweets themselves, instead of blaming or attacking tweet posters, the frequencies of negative emotion and swear words in unhelpful notes are hoped to be lower. In addition, the study also demonstrates the potential of integrating the crowdsourced approach into a broader toolkit for mitigating misleading information. For one thing, the experience garnered through Community Notes can offer valuable practical insights to other online platforms, despite the imperfection in the norms and structures for now. It's important to recognize that differences such as user groups and platform mechanisms should also be taken into account when generalizing these insights (Vraga and Bode, 2018). For another, crowdsourced debunking as part of infodemic management, it's necessary to explore its intersection with other efforts. For instance, classification models can identify AI-generated misinformation but exhibit reduced effectiveness when addressing human-generated misinformation (Zhou et al., 2023). These models can preemptively flag AI-generated content, thereby alleviating the burden on crowdsourced debunking efforts.

This study has several limitations that warrant investigation in future research. First, the study solely took notes that were ultimately voted as either helpful or not helpful as examples. However, there are a large number of notes labeled as NEEDS_MORE_RATINGS. Meanwhile, during the 2-week voting and ranking period, notes upvoted as helpful would be temporarily affixed to tweets on X and remain visible until they receive downvotes. This means some may maliciously exploit the window period to influence people's opinions. Constant exposure to debunking attempts of varying shades probably erode the receivers' confidence in the platform, which in turn results in less positive reactions (Mourali and Drake, 2022). Therefore, future studies can broaden the scope of the corpus to evaluate reliability and effectiveness at different stages. Second, while we conducted a linguistic and psycholinguistic assessment of the collected notes, actual audience responses to the notes on X were not taken into account, such as their perceived severity of the crisis, emotional reactions and attitudes toward taking preventive actions. Future studies examining user perceptions can help corroborate the findings in this study.

## 6 Conclusion

In order to assess the reliability of Community Notes from readability and neutrality, the study collected notes voted as helpful or not helpful by users on Community Notes, spanning from its initial pilot phase to the global expansion. The non-parametric Mann-Whitney *U*-test was applied to examine differences between the two groups based on measures of reading ease, analytical thinking, authenticity and affect. Results reveal that both groups exhibit enhanced readability and helpful notes demonstrate greater logical coherence, authenticity and emotional restraint in accordance with the provisions of the user manual, underscoring the reliability of Community Notes. Nevertheless, negative and abusive language as well as A large value range in the unhelpful group imply management challenges faced by Community Notes. Overall, the research enhances the understanding of crowd wisdom in the context of misinformation debunking and infodemic management. Future endeavors could explore the psychological motivations behind volunteering, gaming or manipulating behaviors, investigate strategies to enhance crowdsourced debunking, and consider its intersection with professional efforts and infoveillance from broader perspectives.

## Data Availability

The raw data supporting the conclusions of this article will be made available by the authors, upon request.

## Footnotes

^1^The Birdwatch website is available at https://communitynotes.x.com/guide/en/contributing/examples.

^2^The data is available at https://communitynotes.x.com/guide/en/under-the-hood/download-data.
